# Correction: Jarzynka, S. et al. Combination of High-Pressure Processing and Freeze-Drying as the Most Effective Techniques in Maintaining Biological Values and Microbiological Safety of Donor Milk. *Int. J. Environ. Res. Public Health* 2021, *18*, 2147

**DOI:** 10.3390/ijerph21070822

**Published:** 2024-06-24

**Authors:** Sylwia Jarzynka, Kamila Strom, Olga Barbarska, Emilia Pawlikowska, Anna Minkiewicz-Zochniak, Elzbieta Rosiak, Gabriela Oledzka, Aleksandra Wesolowska

**Affiliations:** 1 Department of Medical Biology, Faculty of Health Sciences, Medical University of Warsaw, 14/16 Litewska St., 00-575 Warsaw, Poland; sylwia.jarzynka@wum.edu.pl (S.J.); kamila.strom@wum.edu.pl (K.S.); o.barbarska@gmail.com (O.B.); anna.minkiewicz@wum.edu.pl (A.M.-Z.); gabriela.oledzka@wum.edu.pl (G.O.); 2 Institute of High Pressure Physics of the Polish Academy of Sciences, ul. Sokolowska 29/37, 01-142 Warsaw, Poland; emilia.pawlikowska@gmail.com; 3 Institute of Human Nutrition Sciences, Warsaw University of Life Sciences-SGGW, Nowoursynowska 159c St., 02-776 Warsaw, Poland; elzbieta_rosiak@sggw.pl; 4 Laboratory of Human Milk and Lactation Research at Regional Human Milk Bank in Holy Family Hospital, Department of Medical Biology, Faculty of Health Sciences, Medical University of Warsaw, 14/16 Litewska St., 00-575 Warsaw, Poland

## Text Correction

There was an error in the original publication [1]. The work contained incorrect phrases describing the tested strains belonging to the *Bacillus cereus* species. The authors performed experiments only on the vegetative cells of these bacteria. By introducing phrases regarding spores, sporulation, or germination, we only intended to indicate that *B. cereus* are bacteria exhibiting such properties. We used these statements inappropriately.

1. A correction has been made to Section 2. Material and Methods, Section 2.6. Microbiological Safety Assessment, paragraph 1:

Moreover, all the samples were tested for the Gram-negative intestinal *Enterobacteriaceae* family, in particular, *Escherichia coli* and vegetative *Bacillus cereus* rods.

2. A correction has been made to Section 3. Results, Section 3.2.2. Microbiological Purity after High-Pressure Processing and Freeze-Drying in Storage Milk Samples, paragraph 2:

After 6 months of storage, the freeze-dried human milk samples inoculated *Bacillus cereus* showed a complete reduction (by 100%) in the growth of vegetative forms of this species.

3. A correction has been made to Section 3. Results, Section 3.2.2. Microbiological Purity after High-Pressure Processing and Freeze-Drying in Storage Milk Samples, paragraph 3:

HPP was the best method for inactivating vegetative forms of the pathogens present in the inoculated milk samples.

4. A correction has been made to Section 4. Discussions, paragraph 2:

HoP has been shown to result in a complete eradication or decrease in the counts of endogenous bacteria, namely *Escherichia coli* and *Staphylococcus aureus*.

5. A correction has been made to Section 4. Discussions, paragraph 3:

As part of our in-house studies, this milk-processing method led to the complete eradication of vegetative *Bacillus cereus* in all the evaluated human milk samples in comparison with the inoculum. The limitation of this project is that we did not investigate the presence of *Bacillus cereus* spores and the impact of HoP and HPP on the spores.

6. A correction has been made to the Acknowledgements Section. The corrected text appears below.

Acknowledgments: The authors would like to express their gratitude to members of the EMBA Working Group on Processing Milk for finding these errors.

7. A correction has been made to Figure 4. In some cases, the abbreviation of the freeze-drying process was “Lio”. The correct abbreviation is “Lyo”. The corrected version of Figure 4 appears below.

**Figure 4:**
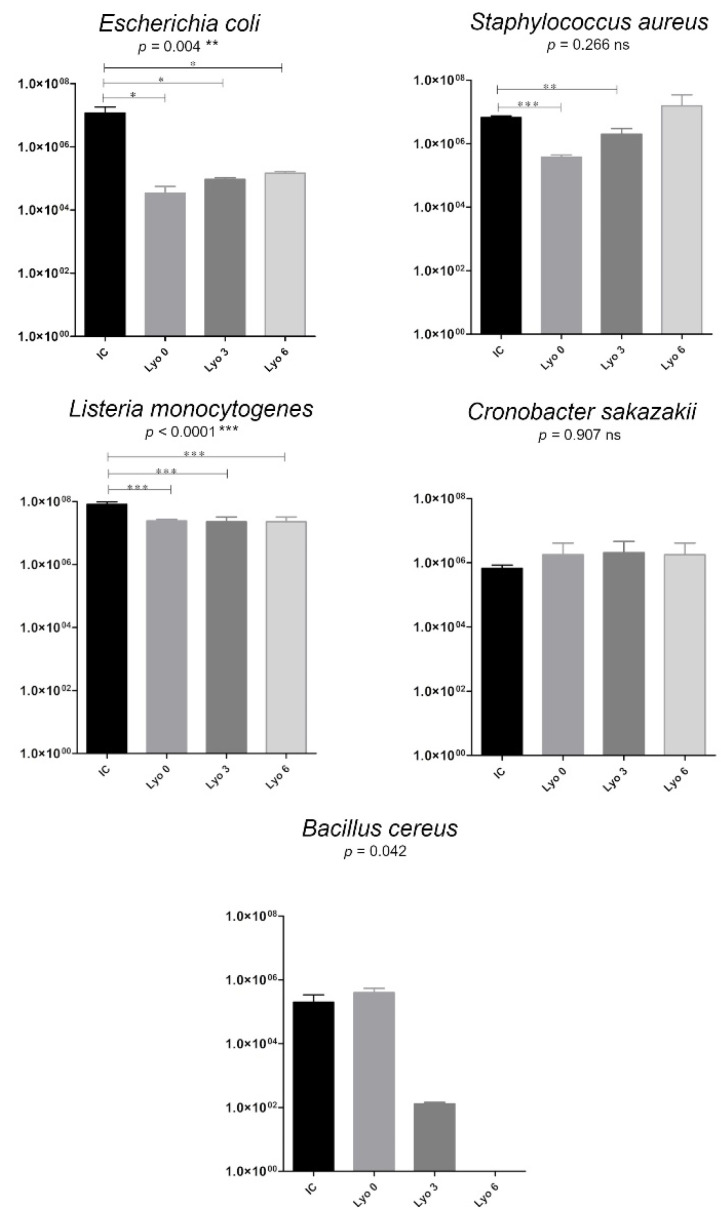
Changes in bacterial growth (colony-forming units (CFU)/mL) in human milk samples inoculated with bacteria strains, freeze-dried, and stored for up to 6 months. The data are presented as mean values with standard deviations. One-way analysis of variance (ANOVA) with post hoc Tukey’s test was used to statistically analyze the data from all replicates; Paired *t*-tests were used for the initial bacterial concentration (IC) and bacterial concentrations after freeze-drying at time 0 (Lyo 0), at 3 months of storage (Lyo 3), and at 6 months (Lyo 6) of storage. Statistically significant differences are marked with asterisks, depending on the degree of significance: *** *p* < 0.0001, ** *p* < 0.001, * *p* < 0.05, whereas non-significant differences (*p* > 0.05) are marked with ‘ns’.

The authors state that the scientific conclusions are unaffected. This correction was approved by the Academic Editor. The original publication has also been updated.
